# BAP31 is involved in T cell activation through TCR signal pathways

**DOI:** 10.1038/srep44809

**Published:** 2017-03-23

**Authors:** Kunwei Niu, Jialin Xu, Yuhua Cao, Yue Hou, Mu Shan, Yanqing Wang, Yang Xu, Mingyi Sun, Bing Wang

**Affiliations:** 1 Institute of Biochemistry and Molecular Biology, College of Life and Health Sciences, Northeastern University, Shenyang 110169, China

## Abstract

BAP31 is a ubiquitously expressed endoplasmic reticulum (ER) membrane protein. The functions of BAP31 in the immune system have not been investigated due to the lack of animal models. Therefore we created a BAP31 conditional knockdown mouse by performing a knockdown of BAP31 in the thymus. In doing so, we demonstrate that the maturation of T cells is normal but the number of T cells is less in the thymus of the knockout mouse. In addition, the spleen and lymph nodes of peripheral immune organs contained a lesser proportion of the mature T cells in the thymus specific BAP31 knockout mice. The BAP31 knockout T cells decreased the proliferation activated by TCR signal pathways. Further studies clarified that BAP31 affects the phosphorylation levels of both Zap70/Lck/Lat of the upstream members and Akt/GSK/Jnk/Erk of the downstream members of TCR signal pathways. Furthermore, BAP31 can regulate the expression of some markers such as CD3/TCRα/TCRβ and some cytokines like IL-2/IFN-γ/IL-6/TNF-α which are important for T cell activation. Taken together, these results demonstrate that BAP31 may play an important role in T cell activation by regulating TCR signaling.

B cell receptor associated protein 31 (BAP31/BCAP31) is an evolutionarily conserved, ubiquitously expressed 28-kDa polytopic integral protein of the endoplasmic reticulum (ER)^1^,^2^,^3^,^4^,^5^, and has been implicated in the ER sorting of diverse client membrane proteins. BAP31 is located head-to-head at Xq28^4^,^6^. The protein contains three predicted transmembrane segments within its amino terminus^1^,^3^. Expression of the BAP31 gene was analyzed from various mouse tissues and cell lines, such as lymphocytes, thymic stromal cells, cerebellar Purkinje neuron bodies, dendrites and thyroid follicular epithelial cells^2^. BAP31 functions as a participants in the transportation of a variety of molecules from the ER to Golgi apparatus, such as newly synthesized IgD, cellubrevin, class I MHC, CD11b/CD18 and protein tyrosine phosphatases like B (PTPLB)^4^,^7^,^8^,^9^,^10^,^11^. BAP31 is involved in the apoptosis and ERAD (Endoplasmic reticulum associated degradation) pathways^12^,^13^,^14^.

T lymphocytes develop in thymus through a series of tightly regulated signaling molecules and can be divided into stages^15^,^16^,^17^. The CD4^-^CD8^−^ double-negative (DN) thymocytes can be divided into four phenotypically distinct subpopulations based on the expression of the CD25 and CD44 markers^18^. The DN cells can further subdivide into the sequential stages DN1 (CD44^**+**^CD25^−^), DN2 (CD44^**+**^CD25^**+**^), DN3 (CD44^−^CD25^**+**^) and DN4 (CD44^-^CD25^−^)^19^,^20^,^21^. Upon TCRβ rearrangement and β-selection, DN cells proliferate and become CD4^**+**^CD8^**+**^ double-positive (DP) thymocytes. DP thymocytes will become either CD4^+^CD8^−^ or CD4^−^CD8^**+**^ single-positive (SP) thymocytes, Finally, SP thymocytes undergo further maturation and selection processes before exiting the thymus as naïve T cells. Naïve T cells (CD44^low^CD62L^hi^) can develop into two main subsets: effector memory (Tem) and central memory (Tcm) cells. Tem cells (CD44^hi^CD62L^Low^) circulate in non-lymphoid organs and can respond rapidly after encountering pathogens or infected cells. In contrast, Tcm cells (CD44^hi^CD62L^hi^) reside in secondary lymphoid organs^22^,^23^,^24^.

The T-cell antigen receptor (TCR) is activated by tyrosine phosphorylation within cytoplasmic regions in the CD3ε, γ, δ and the ζ chain called immunoreceptor tyrosine-based activation motifs (ITAMs). The ITAMs is phosphorylated by the activated Src family kinase Lck. This reaction favors recruitment of the Syk family kinase Zap70, which is, in turn, phosphorylated by Lck. Once Zap-70 has been recruited to the receptor complex and activated, its proximity to the cell membrane allows it to phosphorylate the scaffold protein LAT (linker of activated T cells)^25^, which leads to membrane recruitment of PLC-γ and its phosphorylation and activation by Tec kinases. Activated PLC-γ initiates various downstream signaling molecules, including activation of IκB kinase (IKK), MAP kinases, PI3/AKT and several families of transcription factors, such as NF-κB and AP-1. Consequently, these signaling events induce the production of cytokines, such as IL-2 and IFN-γ, and results in the differentiation, proliferation, and activation of T cells.

A couple of publications indicate that BAP31 participates in class I MHC molecules transportation from the ER to Golgi apparatus^2^,^7^. Since class I MHC molecules play an important role in T cell functions, BAP31 may be involved in T cell development. However, to this day, BAP31 has not been investigated in an immune system for the lack of animal models. Therefore, we generated BAP31 conditional knockout mice and specifically knocked down BAP31 in the thymus. The results showed that BAP31 is involved in T cell activation and proliferation by regulating the expression and phosphorylation of some key members in the TCR signaling pathways.

## Results

### Generation of BAP31 conditional KO mice

**Figure 1:**
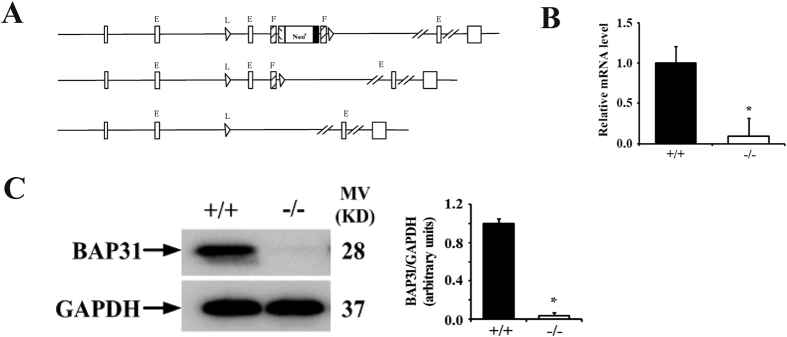
Generation of BAP31 conditional KO mice. (**A**) Diagram of the BAP31 targeting construct and targeted alleles. E, exon; L, *loxP* site; F, FRT site; Neo, neomycin-resistant gene. (**B**) RT-qPCR analysis of genomic RNA from BAP31 conditional KO mice. Total thymocyte RNA from BAP31^−/−^Lck-cre and control mice. (**C**) Western-blot analysis of BAP31 protein expression in total thymocytes from BAP31^f/f^Lck-cre and BAP31^f/f^ mice. Histograms showing BAP31 a relative change in the BAP31^−/−^ (−/−) and BAP31^**+/+**^ (+/+) mice.

To generate BAP31 conditional knockout mice, we constructed a targeting vector with intron 3 of the *BAP31* gene, flanked with two loxp sites (Fig. 1A). A neomycin-resistant gene cassette located within the loxp sites was flanked by two FRT sites (Fig. 1A). Male chimeric mice were bred with FLPeR female mice to delete the neomycin cassette *in vivo*. The BAP31 floxed mice (BAP31^f/f^) were then bred with Lck-cre transgenic mice to induce specific deletion of this gene in T lymphocytes. RT-qPCR analysis of mRNA from total thymocytes of BAP31^f/f^Lck-cre (BAP31^−/−^) and BAP31^f/f^ (BAP31^**+/+**^) mice revealed that BAP31 was deleted in >95% of the thymocytes (Fig. 1B). BAP31 protein expression levels in the thymus of these mice were also reduced >95% (Fig. 1C). These results indicate that Lck-cre expression induced efficient deletion of BAP31 in the developing thymocytes.

### BAP31 is involved in thymus development

**Figure 2:**
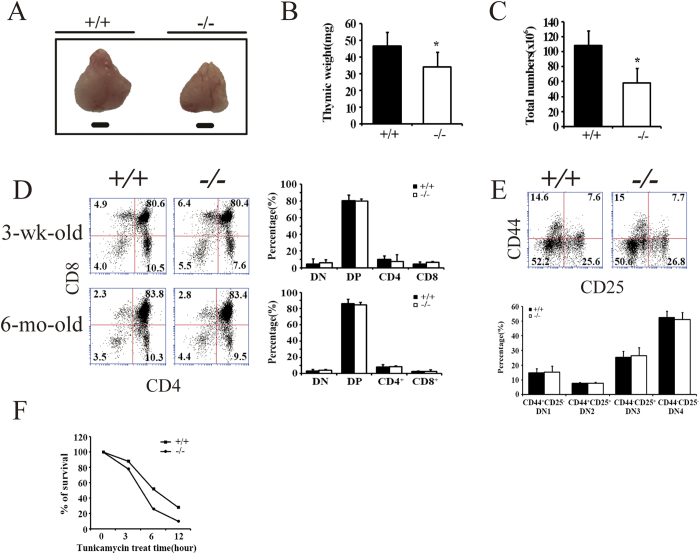
BAP31 involved in thymus development. (**A**) Thymus of BAP31^**+/+**^ (+/+) mice (left, 5 weeks) and BAP31^−/−^ (−/−) mice (right, 5 weeks) from littermates. Horizontal bar stands for 2 mm. (**B**) Thymus weight of BAP31^−/−^ and BAP31^**+/+**^ mice. Histograms depict the thymus weight. S.D. calculated from eight mice. *P < 0.05. (**C**) Thymic cellularity of BAP31^−/−^ and BAP31^**+/+**^ mice. Histograms depict the number of thymus cells. S.D. was calculated from eight mice. (**D**) FACS analysis of thymocytes from young adult and older age BAP31^−/−^ and BAP31^**+/+**^ mice. The percentages of DN (bottom left), CD4 SP (bottom right), DP (top right) and CD8 SP (top left) cells are shown. Histograms showing the numbers of thymic population in the different age BAP31^−/−^ and BAP31^**+/+**^ mice. (**E**) DN thymocytes stained with anti-CD44 and anti-CD25 to identify DN1-DN4 thymocytes from BAP31^−/−^ and BAP31^**+/+**^ mice. Histograms showing absolute numbers of thymic population in the BAP31^−/−^ and BAP31^**+/+**^ mice. (**F**) Percentages of surviving cells after different time of tunicamycin treatment. Total thymocytes were stimulated with 1μg/ml tunicamycin for the indicated time and analyzed for apoptosis by Annexin V and PI staining. The representative data was shown from three separate assays.

The thymus specific BAP31^−/−^ mice had normal growth and development. However, BAP31^−/−^ mice had a significant reduction in thymus size (Fig. 2A), weight (Fig. 2B), and total thymocyte numbers (Fig. 2C) compared with their littermates in the young adult mice (3-, 5- or 8-week-old) and older age (6-mo-old). Thymocyte development was further examined, and the results showed that there was no obvious difference in the DP, CD4SP and CD8SP thymocytes in 3-wk-old and 6-mo-old of BAP31^−/−^ mice (Fig. 2D) or in 5-wk-old and 8-wk-old mice (Supplement Fig. S1). We next examined the expression of CD44 and CD25 by analyzing the CD3^−^CD4^−^ double negative gated thymocytes. The Pro-T cell development as defined by the expression of CD25 and CD44 was comparable in both BAP31^−/−^ and BAP31^**+/+**^ littermate mice. There was no significant effect of BAP31 on the DN1-4 thymic subsets (Fig. 2E), suggesting that the thymocytes in the BAP31^−/−^ mice may develop normally from the DN to the DP, CD4SP and CD8SP stage. To explore whether BAP31 affects T cell survival, freshly isolated thymocytes were treated with Tunicamycin and stained for Annexin V and PI. The result indicated that BAP31 deficient T cells undergo cell death much faster than those of BAP31^**+/+**^ mice (Fig. 2F, Supplement Fig. S2). Collectively, these results demonstrate that the development of CD4^**+**^ and CD8^**+**^SP thymocytes and DN cells was not impaired in BAP31^−/−^ mice. But there was significant reduction in thymus size and total thymocyte numbers.

### BAP31 regulates effector/memory T cells

**Figure 3:**
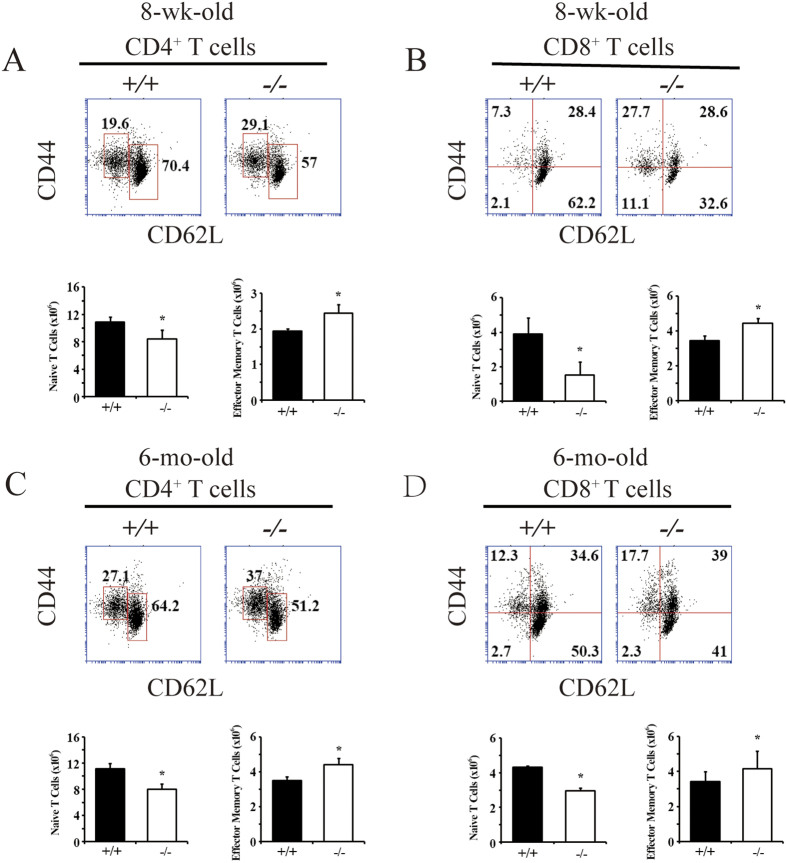
BAP31 regulates effector/memory T cells. Splenocytes were stained for CD4, CD8, CD44 and CD62L. CD4^**+**^ or CD8^**+**^ T cells were gated for CD44 and CD62L analysis. Flow cytometry analyses of the percentage of naïve (CD44^low^CD62L^hi^) and memory (CD44^hi^CD62L^low^) CD4^**+**^ T cells (**A**,**C**) or the percentage of naïve (CD44^low^) and memory (CD44^hi^) CD8^**+**^ T cells (**B**,**D**) in the spleen of young (A and B; 8-wk-old) or older (C and D; 6-mo-old) BAP31^**+/+**^ and BAP31^−/−^ mice. Data are presented as representative plots and summary graphs of the mean ± SD values of three independent experiments.

The peripheral T cell pool is maintained stably under normal conditions, a mechanism termed homeostasis^26^,^27^. Although the majority of peripheral T cells are in a naïve state, characterized by CD44^low^CD62L^hi^ markers, a proportion of memory T cells (CD44^hi^CD62L^low^) exists and increases to high levels at older ages, which is thought to result from homeostatic proliferation induced via TCR stimulation by self-peptide/MHC ligands^26^,^27^. Our results showed that BAP31 deficiency has no effect on the development of CD4^**+**^ and CD8^**+**^SP thymocytes and DN cells, but it is still unknown whether BAP31 plays a role in regulating the homeostasis of peripheral T cells. We addressed this question by analyzing the frequency of naïve and memory T cells in the peripheral lymphoid organs of both young and older mice. We first examined the effector memory population in CD4^**+**^ and CD8^**+**^ T cell gated populations of young adult mice (8-wk-old). Flow cytometric analysis showed that splenic effector memory T cells (CD44^hi^CD62L^low^) in CD4^**+**^ and CD8^**+**^ T cells increased, and the naïve T cell population (CD44^low^CD62L^hi^) was correspondingly reduced (Fig. 3, A,B) in the BAP31^−/−^ mice. Meanwhile, at an older age (6-mo-old), the BAP31^−/−^ mice had similar results in both CD4^**+**^ and CD8^**+**^ T cell populations (Fig. 3, C,D). These results were reproduced in lymph node T cells (data not shown). Thus, BAP31 probably plays an important role in regulating the homeostasis of T cells.

### BAP31 participates in the maturation of T lymphocytes in the peripheral immune organs

**Figure 4:**
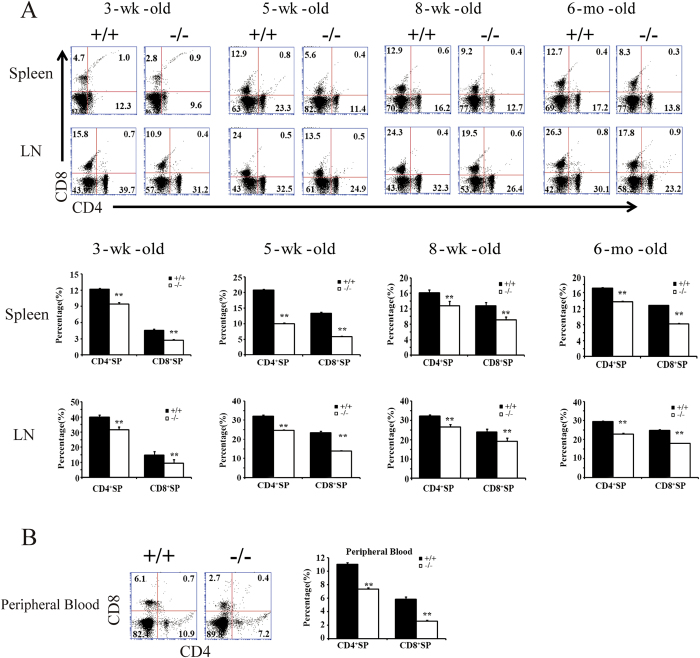
BAP31 participates in the maturation of T lymphocytes in the peripheral immune organs. (**A**) Flow cytometric analyses of CD4 and CD8 expression in the spleen and LN of 3-wk, 5-wk, 8-wk-old and 6-mo-old BAP31^−/−^ and control mice. The percentage for each quadrant is displayed in the appropriate quadrant. Histograms showing absolute numbers of thymic population in the BAP31^−/−^ and BAP31^**+/+**^ mice. S.D. calculated from five mice. **P < 0.01. (**B**) Flow cytometric analyses of CD4 and CD8 expression at CD3^**+**^ gate in peripheral blood BAP31^−/−^ and control mice. Histograms showing absolute numbers of thymic population in the BAP31^−/−^ and BAP31^**+/+**^ mice. S.D. calculated from five mice. **P < 0.01.

T cells developed normally in the thymus even if BAP31 was deleted specifically in the thymus (Fig. 2). T cells will further develop to maturation and activation in the peripheral immune organs after leaving the thymus. We first examined the 3-wk-old mice and found that BAP31^−/−^ mice had less CD4^**+**^ and CD8^**+**^ single positive mature T lymphocytes in the spleen and Lymph nodes (LN) (Fig. 4A). The percentages of CD4^**+**^ and CD8^**+**^ cells in 5-wk-old, 8-wk-old and 6-mo-old BAP31^−/−^ mice were higher than those in 3-wk-old mice but still dramatically lower than those in the littermate controls in peripheral organs (Fig. 4A). Meanwhile, the percentages of CD4^**+**^ and CD8^**+**^ cells in peripheral blood were also lower than the controls (Fig. 4B). Recent publications show that BAP31 participates in molecular transport from the ER to Golgi apparatus. To clarify whether BAP31 regulated the expression of class I MHC molecules, we analyzed the expression of class I MHC molecules on CD4^**+**^ and CD8^**+**^SP splenocytes. The result showed that there was no difference in the expression of class I MHC molecules (Supplement Fig.S3). This result was consistent with the previous studies that loss of BAP31 only impaired the export of class I MHC molecules but does not affect the levels of surface class I MHC molecules^7^. Collectively, these results implied that BAP31 may play a role in the efficient development of mature T lymphocytes.

### BAP31 regulates T cell activation

**Figure 5:**
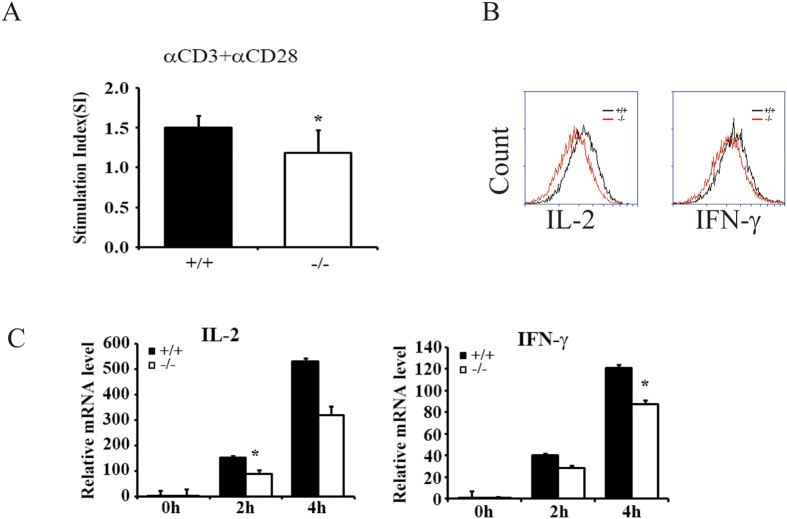
BAP31 regulates T cell activation. (**A**) Proliferation of splenocytes from BAP31^−/−^ and control mice. Total splenocytes were stimulated with plate-bound anti-CD3 plus anti-CD28 for 5 days, and the proliferation was analyzed by MTT. (**B**) Total splenocytes were stimulated with plate-bound anti-CD3 and anti-CD28 for 6 h. Intracellular staining for cytokine production in the total splenocytes was performed. (**C**) RT-qPCR analyses of BAP31^−/−^ and control mice splenocytes stimulated with anti-CD3 and anti-CD28 antibody for the indicated time periods. Shown are representative data from three independent experiments. Error bars reflect S. D.*P < 0.05; **P < 0.01.

To examine the function of BAP31 in regulating T cell activation, we used an *in vitro* approach by stimulating total splenocytes with agonistic antibodies for TCR and followed by measuring T cell responses based on the proliferation and cytokine production. When stimulated with anti-CD3 plus anti-CD28, BAP31-deficient T cells were attenuated in proliferation, as revealed by a relatively lower level of stimulation index than BAP31^**+/+**^ mice (Fig. 5A). The FACS results revealed a profound reduction of the BAP31^−/−^ T cells in producing two major cytokines, IL-2 and IFN-γ (Fig. 5B). Furthermore, the defect of the BAP31^−/−^ T cells in cytokine production was also detected at the mRNA level, indicating impaired induction of the IL-2 and IFN-γ genes (Fig. 5C). These results confirmed the role of BAP31 in regulating T cell activation.

### BAP31 facilitates the up-stream members of TCR signaling

**Figure 6:**
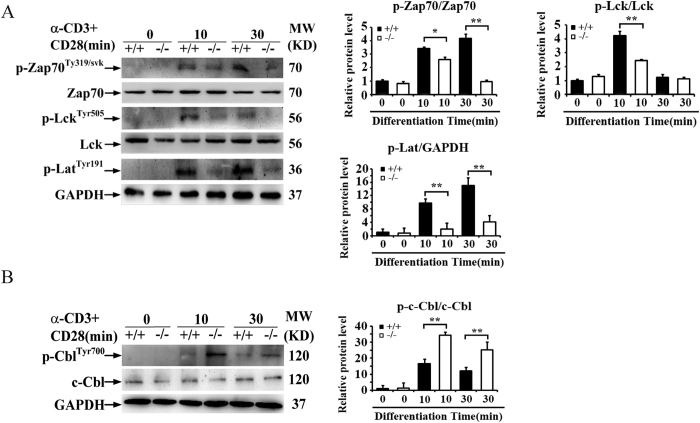
BAP31 facilitates the up-stream members of TCR signaling. (**A**,**B**) Western-blot analyses of the indicated phosphorylated (p-) and total proteins. The lysates from total splenocytes of BAP31^−/−^ and control mice after the stimulation of anti-CD3 and anti-CD28 for the indicated time periods. Histograms showing relative change in the indicated proteins level in the BAP31^−/−^ and control mice. Data in all panels are representative of three independent experiments. Error bars reflect S.D.*P < 0.05; **P < 0.01.

The cell-intrinsic functions of BAP31 in mediating T cell activation and differentiation suggested a role for BAP31 in regulating TCR signaling. TCR signaling initiates from activation of the protein tyrosine kinase Lck, which phosphorylates the TCR-signaling chain CD3ζ, leading to recruitment of the tyrosine kinase Zap70 to the TCR complex, in which Zap70 is phosphorylated and activated by Lck^28^. Activated Zap70 in turn phosphorylates several other signaling molecules, thereby transducing the TCR signal to various downstream signaling events^28^. However, Cross-linking of the TCR and CD28 stimulates proximal signaling events, including tyrosine phosphorylation of Lck, LAT and Zap70. The BAP31 deficiency severely attenuated the phosphorylation of Zap70 and Lck (Fig. 6A). Phosphorylation of the adaptor protein Lat, a major downstream target of Zap70, was also inhibited. In addition, ubiquitination is an important mechanism that regulates T cell activation. c-Cbl as an E3 ubiquitin ligases, has been shown to negatively regulate TCR-CD28 signaling. The BAP31 deficiency severely increased the phosphorylation of c-Cbl (Fig. 6B). These results suggest that BAP31 may facilitate TCR signaling.

### BAP31 facilitates the down-stream members of TCR signaling

**Figure 7:**
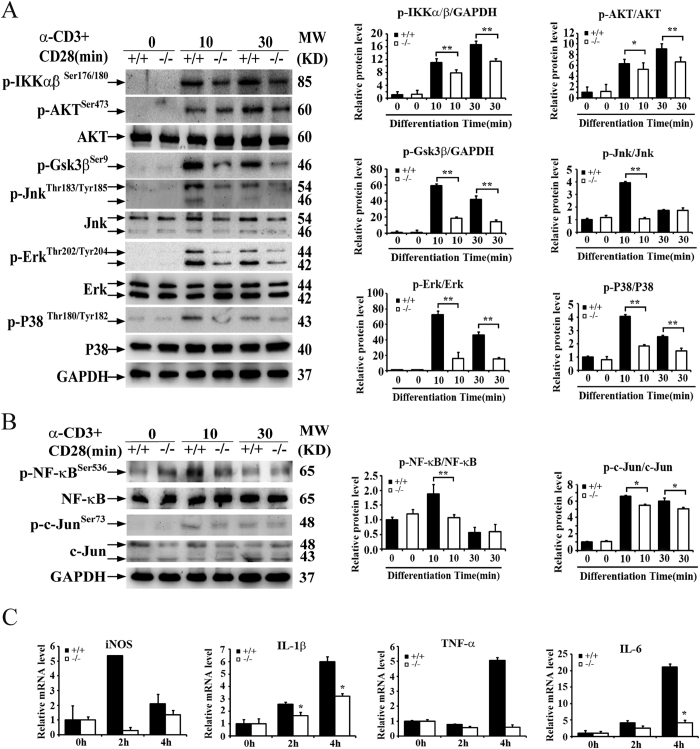
BAP31 facilitates the down-stream members of TCR signaling. (**A**) Western-blot analyses of the indicated phosphorylated (p-) and total proteins. The lysates from total splenocytes of BAP31^−/−^ and control mice after the stimulation of anti-CD3 and anti-CD28 for the indicated time periods. Histograms showing relative change in the indicated proteins level in the BAP31^−/−^ and control mice. (**B**) WB analysis of the indicated transcription factors. Histograms showing relative change in the indicated proteins level in the BAP31^−/−^ and BAP31^**+/+**^ mice. (**C**) RT-qPCR analyses of BAP31^−/−^ and control mice splenocytes stimulated with anti-CD3 and anti-CD28 antibodies for the indicated time periods. Data in all panels are representative of three independent experiments. Error bars reflect S.D.*P < 0.05; **P < 0.01.

As the down-stream members of TCR signaling, NF-κB, PI3/AKT and MAPK are also activated in the T-cell receptor signaling pathways. The TCR-CD28 co-stimulation triggers cascades of signaling events, which regulate the T cell activation^29^. The activation of NF-κB is based on the phosphorylation-induced, proteasome-mediated degradation of IκB. Activation of IKK depends upon phosphorylation at Ser177 and Ser181 in the activation loop of IKKβ (Ser176 and Ser180 in IKKα), which causes conformational changes, resulting in kinase activation^30^. Akt is involved in many cellular processes including metabolism, proliferation, cell survival, growth and angiogenesis. Akt is activated by the phosphorylation at Ser473. Furthermore Akt regulates the storage of glucose by phosphorylating GSK-3 on a serine residue at the N-terminus (GSK-3βSer9), resulting in inhibition of its kinase activity^31^. Extracellular signal regulated kinase1/2 (Erk1/2), C-Jun N-terminal kinase (JNK) and p38 are members of mitogen-activated protein kinase (MAPK) superfamily. The phosphorylation sites of Erk^Thr202/Tyr204^, Jnk^Thr183/Tyr185^ and P38^Thr202/Tyr204^ regulate cellular processes including proliferation, cell survival and growth^32^. We therefore examined the activation of multiple downstream kinases and transcription factors, and found that the BAP31 deficiency attenuated the TCR-CD28-stimulated phosphorylation of several downstream signaling molecules, including IKKα/β, Akt, GSK3β, and MAP kinases in total T cells (Fig. 7A). Consistently, the loss of BAP31 inhibited the activation of two major downstream transcription factors involved in T cell activation, NF-κB and AP-1 (Fig. 7B). Meanwhile, the levels of cytokine production were profoundly decreased in the BAP31^−/−^ (Supplement Fig. S4). A similar result was detected at the mRNA level, which indicated the impaired induction of the iNOS, IL-1β, TNF-α and IL-6 genes (Fig. 7C). The requirement of BAP31 for the activation of multiple downstream pathways suggested a role for BAP31 in the regulation of TCR-proximal signaling events.

### BAP31 facilitates the TCR signaling by regulating the expression of some T cell activation makers

**Figure 8:**
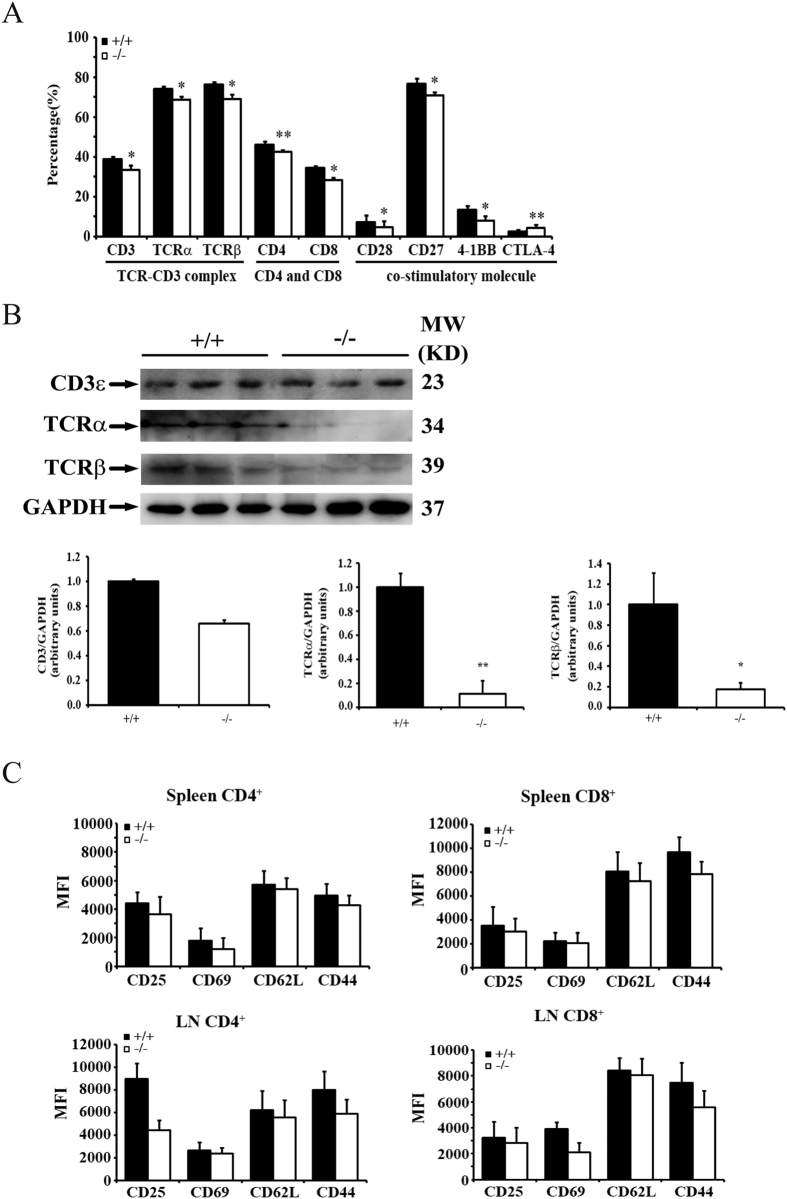
BAP31 facilitates TCR signaling by regulating the expression of T cell activation makers. (**A**) FACS analyses of surface molecule expression on CD3^**+**^ T cells from BAP31^−/−^ and control mice (8-wk-old). Numbers for CD3, CD4, CD8, TCRα, TCRβ, CD28, CD27, 4-1BB and CTLA-4 staining represent the percentages of gated cells. (**B**) Western-blot analyses of the indicated total proteins. The lysates from total splenocytes of BAP31^−/−^ and control mice (8-wk-old). Histograms showing relative change indicated proteins level in the BAP31^−/−^ and control mice. (**C**) FACS analyses of the expression of surface molecule CD4^**+**^ and CD8^**+**^ T cells from BAP31^−/−^ and control mice (5-wk-old). Numbers for CD25, CD69, CD62L and CD44 staining represent the MFI of gated cells. Data in all panels are representative of three independent experiments. Error bars reflect S.D.*P < 0.05; **P < 0.01.

BAP31 is involved in the ER/Golgi traffic of some membrane proteins and unfolded protein degradation. To clarify whether BAP31 regulated the expressions of T cell membrane proteins, we gated the CD3 positive splenocytes to analyze the expression of TCRα, TCRβ, CD4, CD8, and some T cell active co-stimulatory molecules such as CD28, CD27, 4-1BB and an inhibitory co-stimulatory receptor CTLA-4. The results show that these membrane proteins in BAP31^−/−^ mice were expressed in relative lower levels as detected by FACS except for CTLA-4 that was expressed in relative higher levels (Fig. 8A). Similarly the expression levels of CD3, TCRα and TCRβ decreased as detected by Western blot (Fig. 8B). We found that BAP31 regulates T cell activation. Therefore, we gated the CD4^**+**^ and CD8^**+**^ single positive mature cells from spleen or LN to quantify the expression of T cell activation markers CD25, CD69, CD62L and CD44. The results showed that CD4^**+**^ and CD8^**+**^ single positive T cells in BAP31^−/−^ mice expressed lower levels of T cell activation markers (Fig. 8C, Supplement Fig. S5). The above results show that loss of BAP31 decreased the surface expression of TCRα and TCRβ. In addition, our results also showed that BAP31 has a crucial role in regulating the phosphorylation of Zap70. Therefore, we investigated whether BAP31 binds to TCRα, TCRβ and Zap70 in T cells. The results showed that BAP31 and TCRα, TCRβ, Zap70 did not form a detectable complex in both resting and activated T cells (Supplement Fig. S6).

## Discussion

B cell receptor associated protein 31 (BAP31/BCAP31), a member of the B cell receptor associated protein (BAP) family, was first discovered due to its selective binding with mIgD. BAP31 is a ubiquitously-expressed membrane protein of the endoplasmic reticulum (ER). Functionally, BAP31 participates in transportation of a variety of molecules from the ER to Golgi apparatus, such as newly synthesized IgD, cellubrevin, class I MHC molecules, 4-spanning transmembrane super family (TM4SF) of protein^4^,^7^,^8^,^9^,^10^. Class I MHC molecules play an important role in the process of T lymphocyte development. However, the role of BAP31 in T lymphocyte development and function has not been investigated due to lack of BAP31 animal models. Therefore, we generated a BAP31 conditional KO mouse. The Lck proximal promoter becomes most effective in thymocytes starting from the DN3 stage or later^33^,^34^,^35^,^36^,^37^,^38^. When Lck-cre transgene was introduced, the effective deletion of target genes started from the DN3 stage and the obvious deletions were often observed from the DN4 stage and on ward^29^.

Our results show that BAP31^−/−^ mice had a significant reduction in the thymic weight, size and total numbers. Interestingly, in BAP31^−/−^ mice, the proportion of CD4^**+**^ and CD8^**+**^ conventional T cells was not affected by lack of BAP31 in thymus. Similarly there was no significant effect of BAP31 deletion on the DN1-4 thymic subsets. We conjectured that the thymic weight and total numbers reduced, probably due to the increased susceptibility to apoptosis in BAP31^−/−^ mice. In addition, we found that BAP31 plays a role in regulating the homeostasis of peripheral T cells. The results showed that effector/memory T cells (CD44^hi^CD62L^low^) in CD4^**+**^ and CD8^**+**^ T cells increased in the BAP31 deficient mice, and the naïve T cell population (CD44^low^CD62L^hi^) correspondingly reduced. The detailed molecular mechanisms of how BAP31 regulates homeostasis of peripheral T cells await further investigation.

By further analyzing the function of T lymphocyte in BAP31 conditional KO mice, we found that BAP31 played an important role on the efficient function of mature T lymphocytes. The results indicated that BAP31 deficiency resulted in significant lack of mature T lymphocytes in the spleen and LN. Recent studies showed that BAP31 participated in class I MHC molecules transportation from the ER to Golgi apparatus. Our result was consistent with the previous study showing that loss of BAP31 only impairs the export of class I MHC molecules and not the surface class I MHC molecules level^7^,^39^.

Our funding suggested that BAP31 is probably involved in T lymphocyte activation and proliferation. FACS and RT-qPCR revealed a profound reduction of the BAP31^−/−^ T cells in producing two major cytokines IL-2 and IFN-γ, which may be the reason for BAP31’s function in regulation of T cell activations.

Naïve T cell activation is initiated by the engagement of the TCR by a foreign antigen in the context of MHC molecules and also requires ligation of co-stimulatory molecules, primarily CD28. The TCR-CD28 co-stimulation triggers cascades of signaling events, which regulate the initial activation^25^. Our subsequent evidence revealed that a decrease in activation of some key signaling events, including phosphorylation of Zap70, Lck and LAT upon the stimulation of TCR by anti-CD3 plus anti-CD28 Abs. Ubiquitination plays an important role in the regulation of T cell activation. c-Cbl is known to negatively regulate TCR signaling by targeting TCR-signaling molecules for ubiquitin-dependent degradation^40^. Our data indicate that deficiency of BAP31 increased the phosphorylation of c-Cbl, which was consistent with present report that c-Cbl is a negative regulator of TCR. Furthermore, stimulation of T cells with anti-CD3/CD28 Abs results in a decreased phosphorylation of several downstream signaling molecules, including IKKα/β, AKT, GSK-3β and MAPK kinases in total T cells. Consistently, the deficiency of BAP31 inhibited the activation of the major downstream transcription of NF-κB-p65 and C-Jun, the factors involved in T cell activation. These results suggest that BAP31 is required for TCR signaling in conventional T cells.

BAP31 is involved in the ER/Golgi traffic of some membrane proteins and unfolded protein degradation. To clarify how BAP31 regulated the expressions of TCR signaling, we examined the membrane proteins on the cell surface of mature T cells. The results show that these membrane proteins in BAP31^−/−^ mice were expressed in relative lower levels as detected by FACS except for CTLA-4 that was expressed in relative higher levels. Meanwhile, our results indicate that some T cell activation markers CD25/CD69/CD44/CD62L expressed lower levels in BAP31^−/−^ mice. Since AP-1 regulates the expression of CD69 and CD44, BAP31 may affect the expression of CD69 and CD44 by inhibiting the activation of AP-1^41^. To establish whether BAP31 directly associates with the TCR complex, an immunoprecipitation of the TCR was performed. Our results demonstrate that BAP31 facilitates the up-stream and down-stream members of TCR signaling by affecting membrane protein expression.

In a word, we first indicate that BAP31 plays an essential role in T lymphocytes activation by regulating TCR signaling. Our BAP31 conditional KO mice provide a good model to dissect the roles of BAP31 in the immune system.

## Methods

### Generation of BAP31 conditional KO mice

To generate mice specifically lacking BAP31 in T lymphocytes, we constructed a targeting vector with intron3, flanked with two loxp sites. A neomycin resistant gene cassette located within the loxp sites was flanked by two FRT sites. The targeting construct was electroporated into embryonic stem (ES) cells derived from C57BL/6 mice. Male chimeric mice were bred with FLPeR^42^ female mice to delete the neomycin cassette *in vivo*. BAP31^f/+^ mice were then bred with Lck-cre transgenic mice^43^ to generate BAP31^fl/fl^Lck-cre, BAP31^fl/+^Lck-cre and BAP31^fl/fl^ mice. Age-matched, gender same and littermates BAP31^fl/fl^Lck-cre and BAP31^fl/fl^ were used throughout the experiments. All mice were housed under specific pathogen-free conditions at room temperature under a 12 h light/dark cycle, and fed with mouse nutritional food. The present study was approved by the Institutional Animal Care and Use Committee of Northeastern University, and all mice were treated in accordance with the Guide for the Care and Use of Laboratory Animals.

### Fluorescence-activated cell sorter (FACS) analysis of T lymphocytes

Single cell suspensions of the thymus, spleen, and lymph nodes were lysed of Red Blood Cells, the cells were resuspended in 100 μl of 2% BSA in PBS incubated on ice for 10 min, followed by biotinylated mAb, FITC-streptavidin, and PE-, Precpcy5.5-, or APC-labeled mAb were added to the cells which were then incubated on ice water bath at 4 °C for 45 min, and washed with PBS containing 2% BSA. A total of 10^4^ cells events in the gate were collected and analyzed using Fortessa or Accuri C6 and software (BD. Inc). All fluorescence-labeled antibodies, including anti-CD3, −CD4, −CD8, −CD25, −CD69, −CD44, −CD62L, −TCRα, −TCRβ, −CD28, −CD27, 4-1BB and CTLA-4 were obtained from BD Biosciences. Apoptotic cells were determined by Annexin V and PI staining using an Annexin V-FITC kit (BD. Inc) following the manufacturer’s protocol.

### Cytokines measurement using FACS

Single cell suspensions of spleen were lysed of Red Blood Cells, subsequently splenocyte were stimulated with plate-bound anti-CD3 5 μg/ml (eBioscience, Inc) and anti-CD28 2 μg/ml (eBioscience, Inc) in the presence of Leukocyte Activation Cocktail, with BD GolgiPlug™ (BD. Inc) for 6 hours. The collected cells were resuspended in 100 μl of 2% BSA in PBS incubated on ice for 10 min, fixed in 1 ml 2% paraformaldehyde at 4 °C for 1 hour. The cells were resuspended in 100 μl of 0.1% saponin (Sigma, Inc) to which 1:200 each of purified anti-IL-2 rabbit antibody (santa cruz, Inc) were added followed by incubation with 0.5 μg of FITC-conjugated goat anti-rabbit IgG (Proteintech, Inc). The Splenocytes were stained with PerCP/Cy5.5 anti-mouse IFN-γ (Biolegend, Inc) then incubated on ice water bath at 4 °C for 45 min, and washed with PBS containing 2% BSA. The cells were collected and analyzed using Fortessa or Accuri C6 and software (BD, Inc).

### Reverse transcription and quantitative real-time PCR (RT-qPCR)

Total RNA was prepared from the indicated cells using TRIzolreagent (Thermo Fisher Scientific) according to the manufacturer’s instructions. Two microgram of total RNA was converted to cDNA using GoScript™ Reverse Transcriptase (promega, Inc). RT-qPCR was performed using a CFX96 Touch™ Real-Time PCR Detection System (Bio-Rad Laboratories). GoTaq^®^ qPCR Master Mix (promega, Inc) was used according to the manufacturer’s instructions and using the following conditions: 95 °C for 2 min, 95 °C for 15 sec, and 60 °C for 60 sec for 40 cycles. The results were analyzed according to the 2^−ΔΔCq^ formula. The gene specific primers (all for mouse genes) are listed (Table S1).

### Cell proliferation assays

Splenocytes were stimulated with plate-bound anti-CD3 5 μg/ml and anti-CD28 1 μg/ml for the final concentration. Unstimulated cells were grown as controls. After 5 days, cells were added with the MTT (3-(4, 5-dimethylthiazol-2-yl)−2, 5-diphenyltetrazolium bromide) 5 mg/ml (10 μl/sample) and continue incubation for 4 hour at 37 °C. The cell suspension from 96 cell plates were collected into 1.5 ml eppendorf tube and centrifuged for 3500 rpm/5 min. The supernatant was discarded and the precipitate was resuspended in 100 μl/sample DMSO (Dimethyl sulfoxide). By the SYNERGY/H1, read the 570 OD value (optical density). Results of Evaluation: SI (Stimulation index) = CD3 + CD28 OD average value/control OD average value.

### Western blot assay

Single cell suspensions of spleen were lysed of Red Blood Cells from 6- to 8-week-old mice. The cells were stimulated with plate-bound anti-CD3 10μg/ml and anti-CD28 1 μg/ml at a final concentration for indicated time. Unstimulated cells were grown as controls. The cells were resuspended in buffer RAPI (50 mM Tris-Hcl, pH 7.4, 0.2% TritonX-100, 0.1% SDS, 150 mM Nacl, 2 mM EDTA, 50 mM NaF, 10% Na-deoxycholate, 2.5 mM sodium pyrophosphate decahydrate, 1 mM sodium orthovanaldate, 1 mM phenylmethanesulfony fluoride) at 4 °C for 45 min, then centrifuged at 12000 g/15 min. To the supernatant, 5× sodium dodecyl sulfate (SDS) sample buffer was added and boiled. Proteins were separated by 10% SDS-polyacrylamide gel, electrophoresis and transferred to a polyvinylidenedifluoride membrane (Milipore, Inc). Washed with Tris buffered salin-0.5% Tween 20 (TBS-T) and blocked with 5% nonfat milk-TBS-T at room temperature for 1 hour followed by incubation with primary antibodies overnight. Anti-rabbit polyclonal antibody was from CST. The signal was detected with ECL Western Blotting Detection Reagents (Milipore, Inc). The antibody source and dilution are listed (Table S2).

### Statistical analysis

All experimental data are reported as means ± experimental standard deviations (SD). Student’s t-test was used to determine the significance of the results (significance: *P < 0.05; **P < 0.01).

## Additional Information

**How to cite this article:** Niu, K. *et al*. BAP31 is involved in T cell activation through TCR signal pathways. *Sci. Rep.* **7**, 44809; doi: 10.1038/srep44809 (2017).

**Publisher's note:** Springer Nature remains neutral with regard to jurisdictional claims in published maps and institutional affiliations.

## Supplementary Material

Supplementary Information
