# Supplementary material for: BAP31 is involved in T cell activation through TCR signal pathways
Source: Sci Rep. 2017 Mar 23;7:44809. doi: 10.1038/srep44809 (PMC5363085; doi:10.1038/srep44809)
Supplement: Supplementary Information [file srep44809-s1.pdf]

Supplementary Material

BAP31 is involved in T cell activation through TCR signal pathways

Kunwei Niu, Jialin Xu, Yuhua Cao, Yue Hou, Mu Shan, Yanqing Wang, Yang Xu, Mingyi Sun & Bing Wang*

Institute of Biochemistry and Molecular Biology, College of Life and Health Sciences, Northeastern University, Shenyang 110169, China. *Correspondence and requests for materials should be addressed to B.W. (wangbing@mail.neu.edu.cn)

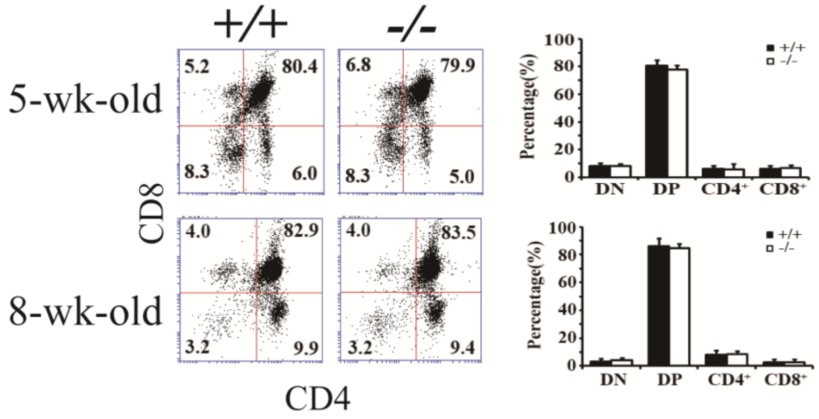

Supplemental Figure S1. **FACS analysis of thymocytes from young adult age BAP31$^{-/-}$ and BAP31$^{+/+}$ mice.** The percentages of DN (bottom left), CD4SP (bottom right), DP (top right) and CD8SP (top left) cells are shown. Histograms showing absolute numbers of thymic population in the different age BAP31$^{-/-}$ and BAP31$^{+/+}$ mice.

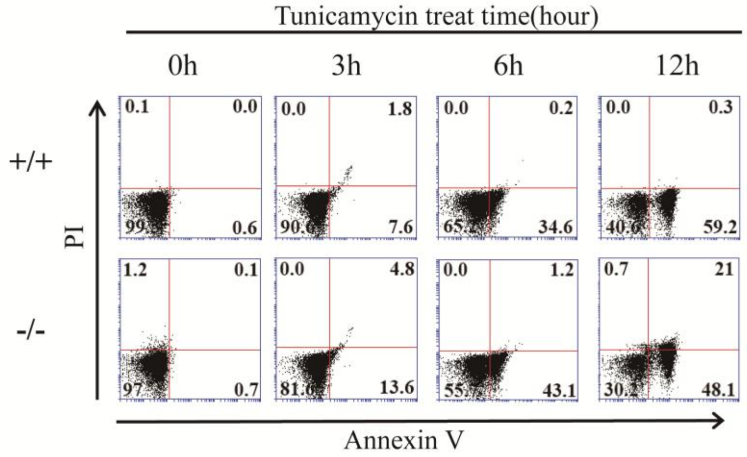

Supplemental Figure S2. Percentages of surviving cells after different time of tunicamycin treatment. Total thymocytes were stimulated with 1μg/ml tunicamycin for the indicated time and analyzed for apoptosis by Annexin V and PI staining. The gate setting distinguished between living (bottom left), necrotic (top left), early apoptotic (bottom right) and late apoptotic (top right) cells.

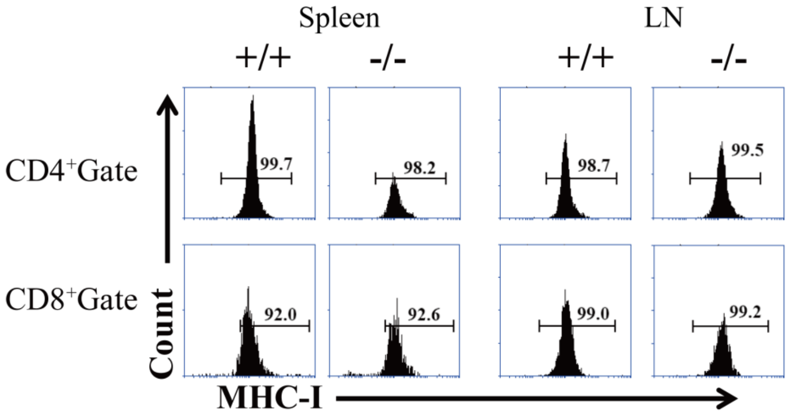

Supplemental Figure S3. BAP31 deficiency dose not decrease the levels of surface class I MHC molecules. Flow cytometric analyses of MHC-I expression at CD4$^+$ gate or CD8$^+$ gate in spleen and LN BAP31$^{-/-}$ and control mice. The percentages of MHC-I are shown.

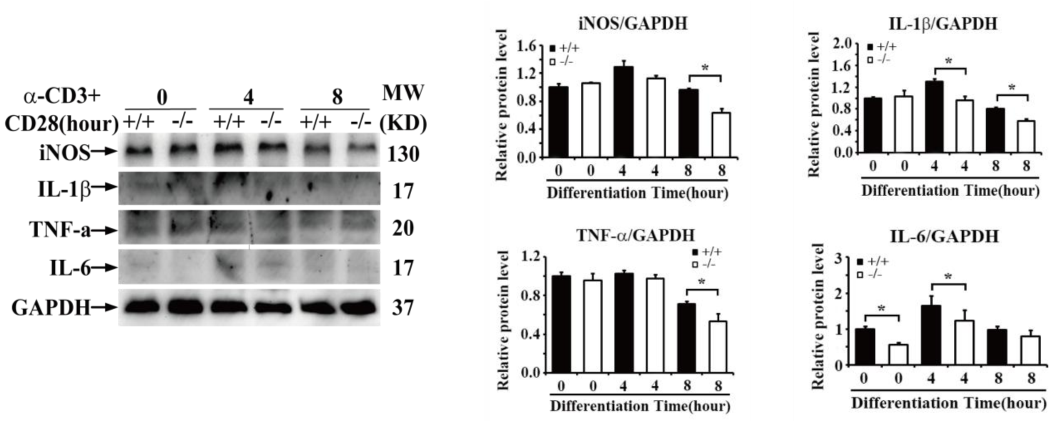

Supplemental Figure S4. WB analysis of the indicated cytokine. Data in all panels are representative of three independent experiments. Error bars reflet S.D.*, P <0.05; **, P <0.01.

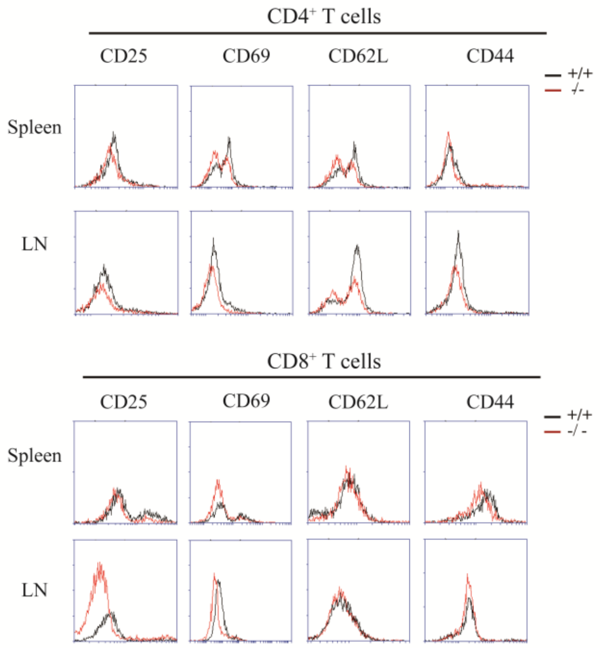

Supplemental Figure S5. FACS analyses of the expression of surface molecule $CD4^+$ and $CD8^+$ T cells from $BAP31^{-/-}$ and control mice (5-wk-old). Numbers for CD25, CD69, CD62L and CD44 staining represent the percentages of gated cells.

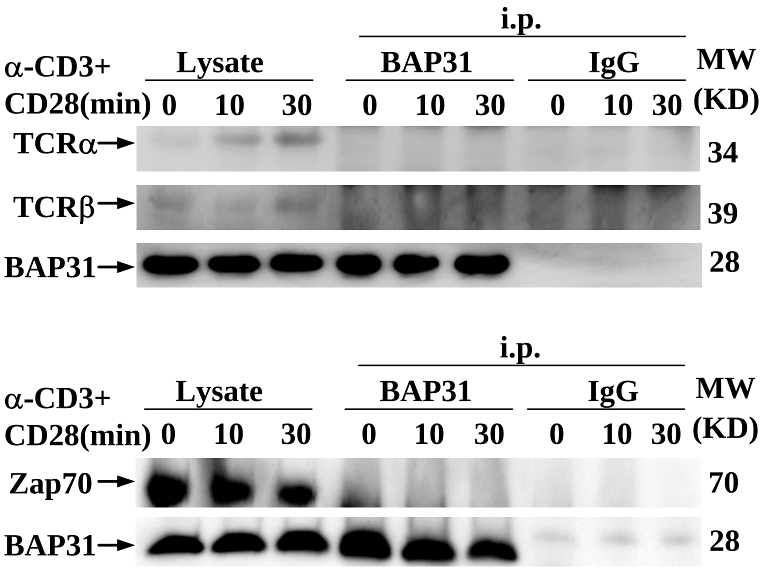

Supplemental Figure S6. IP analyses BAP31 Interact with TCRα, TCRβ and Zap70. Total cell lysates (1% NP40 buffer) from splenocyte cells were subjected to immunoprecipitation (i.p.) with mice preimmune or anti-BAP31, and the precipitates were probed by blotting with anti-TCRα and anti-TCRβ (upper panel) with anti-BAP31 (lower panel), anti-Zap70 (upper panel) with anti-BAP31 (lower panel).

Supplemental Table S1. Primer sequences for RT-qPCR

Primer	Sequence
18s	F: 5’-AGTCCCTGCCCTTTGTACACA-3’
	R: 5’-CGATCCGAGGGCCTCACTA-3’
BAP31	F: 5’-GCCACCTTCCTCTACGCAG-3’
	R: 5’-TGCCATAGGTCACTACCAACTC-3’
IL-2	F: 5’-TGAGCAGGATGGAGAATTACAGG-3’
	R: 5’-GTCCAAGTTCATCTTCTAGGCAC-3’
IFN-γ	F: 5’-ATGAACGCTACACACTGCATC-3’
	R: 5’-CCATCCTTTTGCCAGTTCCTC-3’
iNOS	F: 5’-GGCAAACCCAAGGTCTACGTT-3’
	R: 5’-GAGCACGCTGAGTACCTCATTG-3’
IL-1β	F: 5’-GACCTTCCAGGATGAGGACA R-3’
	R: 5’-AGCTCATATGGGTCCGACAG-3’
TNF-α	F: 5’-GCTACGACGTGGGCTACAG-3’
	R: 5’-GCTACGACGTGGGCTACAG-3’
IL-6	F: 5’-AGTTGCCTTCTTGGGACTGA-3’
	R: 5’-CAGAATTGCCATTGCACAAC-3’

Supplemental Table S2. List of primary antibodies used in this study

Antibody	Cat#	source	dilution
GAPDH	2118	CST	1:4000
Zap70	3165	CST	1:1000
phospho-Zap70	2717	CST	1:1000
Lck	2752	CST	1:1000
phospho-Lck	2751	CST	1:1000
phospho-LAT	3584	CST	1:1000
Akt	4691	CST	1:1000
phospho-Akt	4060	CST	1:1000
phospho-GSK3β	5558	CST	1:1000
phospho-IKKα/β	2697	CST	1:1000
Jnk	9252	CST	1:2000
phospho-Jnk	4668	CST	1:2000
Erk	4695	CST	1:2000
phospho-Erk	4370	CST	1:2000
P38	8690	CST	1:2000
phospho-P38	4511	CST	1:2000
NF-κB	8242	CST	1:1000
phospho-NF-κB	3033	CST	1:1000
C-Jun	9165	CST	1:1000
phospho-C-Jun	3270	CST	1:1000
c-Cbl	2197	CST	1:500
phospho-c-Cbl	SC-26140-R	Santa Cruz	1:500
iNOS	13120	CST	1:1000
TNF-α	11948	CST	1:1000
CD3-ε (M-20)	sc-1127	Santa Cruz	1:500
TCRα (H-142)	sc-9100	Santa Cruz	1:50
TCRβ (H-197)	sc-9101	Santa Cruz	1:100
